# An integrated vitamin E-coated polymer hybrid nanoplatform: A lucrative option for an enhanced *in vitro* macrophage retention for an anti-hepatitis B therapeutic prospect

**DOI:** 10.1371/journal.pone.0227231

**Published:** 2020-01-10

**Authors:** Mohamed Hamdi, Hend Mohamed Abdel-Bar, Enas Elmowafy, Khuloud T. Al-Jamal, Gehanne A. S. Awad

**Affiliations:** 1 Department of Pharmaceutics, Faculty of Pharmacy, University of Sadat City, Sadat City, Egypt; 2 Department of Pharmaceutics and Industrial Pharmacy, Faculty of Pharmacy, Ain Shams University, Sadat City, Egypt; 3 Institute of Pharmaceutical Science, Faculty of Life Sciences & Medicine, King’s College London, England, United Kingdom; Chemistry: LAQV REQUIMTE, PORTUGAL

## Abstract

A platform capable of specifically delivering an antiviral drug to the liver infected with hepatitis B is a major concern in hepatology. Vaccination has had a major effect on decreasing the emerging numbers of new cases of infection. However, the total elimination of the hepatitis B virus from the body requires prolonged therapy. In this work, we aimed to target the liver macrophages with lipid polymer hybrid nanoparticles (LPH), combining the merit of polymeric nanoparticles and lipid vesicles. The hydrophilic antiviral drug, entecavir (E), loaded LPH nanoparticles were optimized and physicochemically characterized. A modulated lipidic corona, as well as, an additional coat with vitamin E were used to extend the drug release enhance the macrophage uptake. The selected vitamin E coated LPH nanoparticles enriched with lecithin-glyceryl monostearate lipid shell exhibited high entrapment for E (80.47%), a size ≤ 200 nm for liver passive targeting, extended release over one week, proven serum stability, retained stability after refrigeration storage for 6 months. Upon macrophage uptake *in vitro* assessment, the presented formulation displayed promising traits, enhancing the cellular retention in J774 macrophages cells. *In vivo* and antiviral activity futuristic studies would help in the potential application of the ELPH in hepatitis B control.

## 1. Introduction

Lately an increased alertness about the role of hepatic macrophages in viral hepatitis has been recognized [1, 2]. Being members of the reticuloendothelial system, they have been acknowledged as key participants in the prevention of the disease progression [2]. Delivery strategies using nanotherapeutics to target the macrophages had been well specified [3]. The nanocarriers should possess a defined size, with a targeted engineered modified surface to facilitate both passive and active (cellular targeting) liver targeting [4].

Hepatitis B virus (HBV) infection accounts for increased deaths worldwide, due to the risk of cirrhosis, hepatocellular cancer, portal hypertension and liver failure [5]. To date, the antiviral therapies, have failed to realize complete removal of the virus from the body and treatment prolonged duration, without the possible emergence of drug resistance [6, 7]. Due to its high lethality, researchers advanced that in the future, good therapy of HBV should be a composite treatment build up from nucleot(s)ide analogs, immunostimulants and possibly curative vaccines.

Among the antiviral nucleot(s)ide analogues, entecavir (E), has been highly recommended due to various merits: (i) potency, (ii) low resistance, and (iii) low systemic toxicity [8–10]. In a very recent multicenter cohort study conducted by Chinese researchers, E monotherapy offered evidence of lowered virological breakthrough and heightened HBV-DNA suppression compared to combination therapy of antiviral agents; lamivudine and adefovir [9]. Zoutendijk and coworkers suggested E monotherapy for 48 weeks [11]. However, the oral administration of E is associated with poor patient compliance and adverse reactions, resulting from intake of E on empty stomach, necessitating the fabrication of sustained release formulation [6]. In this context, long-acting parenteral formulations of E have been well-investigated by various research groups, including liquid crystals [12], lipidic prodrug [13], albumin nanoparticles [8] and PLGA microspheres [6].

Highly optimized carriers with sub-cellular targeting moieties have been opted to enhance hepatic (Kupffer cells and hepatocytes) targeting efficiency for competent antiviral delivery [5]. Fabrication of polymeric nanoparticles and lipid nanocarriers is considered as the most noticeable tool to fulfill such purpose. Polymeric nanoparticles have been employed to provide good hydrophobic drug loading, biodegradability, structural integrity and stability characteristics [14, 15]. On the other hand, the use of lipid nanocarriers such as liposomes is well-consolidated, due to their good biocompatibility as well as the ease of modification by different targeting moieties [16], Besides, up till now, they are the only nanocarriers being approved in clinical applications and scaled-up for industrial production [17]. However, both nanocarriers, when utilized separately, have some limitations due to poor loading of hydrophilic bioactive compounds and uncontrolled drug release from polymeric nanoparticles and drug leakage and instability during storage from lipid nanocarriers [18, 19].

Hence, lipid-polymer hybrid nanoparticles (LPH) was designed, combining the advantages of both polymer- and lipid-based nanoparticles. LPH are archetype of nanocarriers composed of an internal polymeric core enclosed by an outer lipid bioactive shell composed of one or more layers or components [20, 21]. By virtue of their unique structure, LPH have versatile competence to encapsulate different types of payloads such as hydrophilic, hydrophobic drugs or nucleic acids, highlighting promising *in vivo* therapeutic outcomes [22–25]. The concomitance of both polymer and lipid imparts the LPH structural integrity, serum stability, sustained drug release (from the polymer core) and high biocompatibility (from the lipid shell) [21]. Finally, rapid opsonization induced by the lipidic shell resulting in rapid uptake of the particles by the mononuclear phagocyte system (MPS) in the liver and spleen has been previously proven [26, 27].

Different polymers and lipids have been exploited for the fabrication of LPH. PLGA, a synthetic polyester, approved by US FDA and European Medicine Agency for human use has been extensively investigated in the fabrication of LPH [28]. Lecithin (LEC) is a type of phospholipids which are vital components of the cell membrane to conserve membrane fluidity [29]. Generally, phospholipids play a crucial role in solubilization, sustainment of drug release, improvement of therapeutic efficacy and biocompatibility [30, 31].

In light, the aim of this study is to formulate and evaluate a new long-acting macrophage-directed platform for the antiviral agent E. This work provides the first utilization of LPH in E (ELPH) parenteral delivery. The optimized formulae, obtained from Box-Behnken (BBD) statistical design, were subjected to different modulations to prolong the drug release to the maximum. Firstly, lecithin (LEC) lipid shell was admixed with other lipids such as cholesterol (CH) or glyceryl monostearate (GMS) and secondly the NPs were further coated with vitamin E. Both CH and GMS have been reported to control drug release from different carriers [32, 33].

CH is well-reputed neutral lipid commonly used in liposomes, heightening the packing of phospholipid molecules, rigidity and stabilization of liposomes [34]. GMS is a single-chained biocompatible self-assembly amphiphilic monoglyceryl ester, showing significant applications in drug delivery as self-nanoemulsifying drug delivery systems, solid lipid nanoparticles, polymer lipid nanoparticles [35–37]

**Vitamin E**, is a lipid soluble vitamin with antioxidant [38] and immunomodulatory activities [39]. It has been reported to improve the phagocytic potential of macrophage in broilers [40]. Moreover, it has several transport pathways from serum to liver by different vitamin E–binding proteins as α-tocopherol-associated proteins (SEC14L2, SEC14L3, and SEC14L4) and the albumin-related protein, afamin[41, 42].

It is worth mentioning that this investigation assesses for the first time macrophage targeting of vitamin E coated E loaded nanopharmaceuticals, as an indicator of the efficient hepatic therapeutic system, an issue that has not been attempted yet in previously reported E carriers. In this regard, confocal laser scanning microscopy (CLSM) and flow cytometry was performed to evaluate the qualitative and quantitative macrophage retention of ELPH respectively.

## 2. Materials and methods

### 2.1. Materials

Entecavir (E): kindly provided by Mash Premiere for Pharmaceutical Industry, Egypt. Glyceryl monostearate (GMS): purchased from Carl Roth GmbH, Germany. 50/50 DL-lactide/glycolide conjugate, acid terminated (PLGA, with an inherent viscosity midpoint of 0.2 dl/g): kindly supplied from Purac Biomaterials. Lecithin (LEC) soybean (3-*sn*-Phosphatidylcholine ≥99% (TLC) lyophilized powder), cholesterol (CH), vitamin E (α-Tocopherol ≥95.5%), acetonitrile (HPLC grade), ethanol absolute, dimethylformamide (DMF), dimethylsulphoxide (DMSO), 4′,6-Diamidino-2-phenylindole (DAPI), RPMI and fetal bovine serum (FBS): purchased from Sigma–Aldrich Company, UK. Mannitol: purchased from BDH Chemicals Ltd, Poole, UK. Potassium dihydrogen orthophosphate, sodium hydroxide, sodium chloride, potassium chloride, sodium dibasic hydrogen orthophosphate, hydrochloric acid and Tween 80 (polysorbate 80): obtained from Fluka Chemika-BioChemika, Switzerland. Triton^™^ X-100 and 1,1'-Dioctadecyl-3,3,3',3'-Tetramethylindocarbocyanine Perchlorate (DiI): purchased from Fisher Scientific, UK. Vectashield^®^ mounting media was from Vector Labs (UK).

### 2.2 Methods

#### 2.2.1. Determination of entecavir solubility in different lipids

The solubility of entecavir (E) in different single lipids or different mixtures of LEC with CH or GMS was determined as described in supplementary information [43, 44].

#### 2.2.2. Preparation of E LPH

Different formulations ELPH were prepared using modified single-step nanoprecipitation self-assembly method as described elsewhere [14]. Briefly, different amounts of PLGA and E were dissolved in DMF forming the organic phase. According to the output needed from the study, the aqueous phase containing different weights of LEC with or without CH or GMS and Tween 80 (1% w/v) were dissolved in 4%v/v hydroalcoholic solution and heated to 70°C for 15 min. The amounts and concentrations of polymers/lipids in different ELPH formulations are listed in Table 1. Slow dripping of the organic phase into the aqueous phase was done while stirring at room temperature for 2 h. The ratio of organic to aqueous phase was kept at 1:9 v/v. The resultant LPH dispersions were centrifuged at 12000 rpm for 30 min at 4°C and the harvested pellets were re-suspended in PBS (pH 7.4) for further analysis. The optimized ELPH was incubated with vitamin E (1 mL for 2h) to produce EELPH. After soaking, the dispersions was re-centrifuged under the same conditions to remove excess vitamin E. For estimations of entrapment efficiency (EE %) and loading capacity (LC), a fixed volume was removed before centrifugation as a total drug reference. DiI-labelled ELPH and EELPH were prepared by dissolving the dye into the lipid phase at 1% w/w. Freeze-dried ELPH and EELPH were obtained after 48h lyophilization to obtain a free-flowing powder, when required.

**Table 1:** Levels of critical process parameters for the preparation of ELPH using the BBD.

Critical process parameters (Coded independent variables)^a^	Levels		
	Low (-1)	Medium (0)	High (1)
A: PLGA (mg)	5	10	15
B: LEC (mg)	1	2	3
C:Drug (mg)	5	10	15
D:Stirring speed (rpm)	500	750	1000

^a^ The quality attributes and quality target product profiles was PS (< 200 nm) and maximum Entrapment efficiency (EE %).

#### 2.2.3. Experimental design and construction of Box-Behnken (BBD) design

Optimization of ELPH was done using BBD (Design-Expert 9.0.5.2, State-Ease Inc., USA) to construct matrix and explore both the response surfaces and the statistical models [45]. The selected critical process parameters (CPPs) i.e. the independent variables were PLGA, LEC, E amounts and stirring speed which respectively labelled as A, B, C and D. Each variable was tested at three levels; low (-1), medium (0) and high (+1). The particle size (PS) (*Y1*) and entrapment efficiency (EE %) (*Y2*) were the critical quality attributes (CQAs). The ELPH were designed to deliver quality target product profile (QTPP) of PS less than 200 nm and maximum EE%. The defined CPPs and CQAs, as well as the desired QTPP, are listed in Table 1.

The design matrix generated by the software consisted of 29 different runs (Table 2). ANOVA was used for the statistical validation of the polynomial equations generated by Design Expert software. All the responses observed were simultaneously fitted to linear; two-factor interactions (2FI) and quadratic models. The three-dimensional (3-D) response surface plots were constructed by the software and the polynomial equations were authenticated.

**Table 2:** Experimental design matrix of the CPPS and the related CQAs.

Run	Critical process parameters (CPPs)				Critical quality attributes (CQAs)	
	A PLGA (mg)	B LEC (mg)	C Drug (mg)	D Stirring speed (rpm)	Y1 PS (nm)^a^^,^^c^	Y2 EE (%)^b^^,^^c^
1	10	2	10	750	150±2	63.21±2
2	15	2	15	750	215±2	77.63±3
3	10	2	15	500	310±3	72.05±1
4	10	3	10	1000	165±3	74.89±3
5	10	2	10	750	160±2	68.09±4
6	10	2	10	750	161±3	69.81±3
7	10	1	10	1000	137±2	76.61±3
8	10	2	10	750	159±2	68.39±4
9	5	2	5	750	134±2	38.25±3
10	15	2	10	500	285±2	59.61±3
11	15	2	5	750	157±4	38.91±3
12	10	3	15	750	189±2	76.84±2
13	10	1	15	750	196±2	78.59±3
14	5	2	15	750	137±4	68.09±4
15	15	3	10	750	194±2	60.16±5
16	10	2	15	1000	190±3	76.51±3
17	5	2	10	500	269±4	55.87±3
18	10	2	5	1000	125±3	46.86±3
19	5	3	10	750	137±4	76.6±3
20	5	1	10	750	128±3	47.09±2
21	5	2	10	1000	138±4	64.41±3
22	15	2	10	1000	169±2	68.54±3
23	15	1	10	750	139±3	75.74±2
24	10	3	10	500	295±2	63.86±4
25	10	2	5	500	266±3	34.09±2
26	10	3	5	750	188±3	51.09±3
27	10	1	10	500	275±3	60.44±1
28	10	2	10	750	180±3	76.59±2
29	10	1	5	750	135±4	40.98±5

^a^ PS was measured by DLS.

^b^ Calculated as percentage of initial E added, determined directly by HPLC.

^c^ Expressed as mean ± SD (n = 3).

Based on the highest desirability, the design space was created to define the optimum CPPs [46]. Three optimum checkpoints were picked for validation of the chosen domain and equations. The experimental values of the responses were quantitatively compared with that of the predicted values and prediction error (%) were calculated.

#### 2.2.4. Determination of particle size, size distribution and zeta potential (ξ)

The PS (z-average) and size distribution expressed as polydispersity index (PDI) of the prepared ELPH, as well as ξ of the optimized ones, were estimated by dynamic light scattering technique (DLS) with a Nanosizer ZS Series (Malvern Instruments, UK).

#### 2.2.5. Entrapment efficiency and loading capacity

The entrapment efficiency (EE %) was determined directly by measuring the amount of E entrapped inside the LPH. Accordingly, a specified volume of the prepared dispersions (5 mL) was centrifuged at 12000 rpm for 30 min at 4°C and the collected pellets were dissolved into DMF (10 mL). The amount of encapsulated drug was quantified using a previously validated HPLC method (Agilent 1100, Germany, equipped with G 1311A quaternary pump and UV detector (VWD-G1314 A). A reverse phase C18 column (Thermo^®^ BDS, 250X4.6 mm, 5μ) was used at 25°C. The wavelength of the UV detector was set at 254 nm. The flow rate of the mobile phase, composed of acetonitrile and 10 mM phosphate buffer pH 3.5 at a ratio of (80:20), was adjusted at 1 mL/min.

The EE % and LC % were calculated according to the following equations [24]:

$$EE\%=\frac{amount\;of\;entecavir\;inside\;the\;pelletes}{Total\;amount\;of\;entecavir\;added}*100$$ (1)

$$LC\%=\frac{mass\;of\;entecavir\;inside\;the\;pelletes}{Total\;mass\;of\;entecavir\;LPH\;NPs}*100$$ (2)

#### 2.2.6. *In vitro* drug release study

Aliquots of ELPH or EELPH (equivalent to 5mg E) was placed in the presoaked dialysis membrane (cut off: 10,000 Da), diluted with 1mL of PBS (pH 7.4) and mixed with fresh rat serum (FRS) at 50% v/v final concentration [47]. The dialysis membrane method with slight modifications was used [48]. The tightly closed membranes were put in containers filled with 50 mL PBS (pH 7.4) in a thermostatically controlled shaking water bath at 250 strokes/min ±0.1 at 37±0.5°C. At predetermined time intervals, an aliquot of 0.5 mL was withdrawn and replaced. The samples were analyzed using the validated HPLC method and the percentage of E released was calculated.

The release profiles were compared by applying the similarity factor (*f*_2_). Two dissolution profiles were considered similar when the *f*_2_ value is ≥50 [48].

#### 2.2.7. Morphological studies

*2*.*2*.*7*.*1*. *Transmission electron microscope (TEM)*. EELPH was visualized using transmission electron microscope (TEM, Jeol, JEM-1230, Japan). A drop of the optimized EELPH dispersion was deposited on a copper 300-mesh grid, coated with carbon and allowed to stand for 10min after which, any excess fluid was absorbed by a filter paper. Before the examination, one drop of 1% phosphotungstic acid was applied and allowed to dry for 5min.

*2*.*2*.*7*.*2*. *Atomic force microscope*. The 3-D surface profile and topographical image for EELPH were visualized by atomic force microscope (AFM, Wet-SPM 9600, Scanning probe microscope, Shimadzu, Japan.) under normal atmospheric conditions. One drop of the optimized EELPH dispersion was placed on a silicon wafer and allowed for air drying. The measurements were performed using high-resonant-frequency pyramidal cantilevers with silicon probe. The cantilever had a nominal force constant of 0.35–6.06 N/m with a scan speed of 2Hz. The AFM images were analyzed using non-contact mode software [49].

#### 2.2.8. *In vitro* serum stability assay

The *in vitro* stability of EELPH was assessed by recording the PS, PDI and ξ after incubation with 10% and 50% v/v fetal bovine serum (FBS) for 4, 24 and 48 h at 37± 0.5°C [50].

#### 2.2.9. *In vitro* hemolytic assay

Haemolytic activity of the optimized EELPH was assessed using fresh male albino rat’s red blood cells (RBCs). All animal experiments were conducted in agreement with the project license (PBE6EB195) granted by the UK Home Office and in accordance with the U.K. Animals (Scientific Procedures) Act, 1986 and associated guidelines, EU Directive 2010/63/EU for animal experiments. Briefly, blood was withdrawn on heparinized tube from male albino rat (aged 2–3 months, 200g ±10%) tail vein (1 mL from each animal). The blood was centrifuged at 4000 rpm for 10 min to collect the RBCs. The collected RBCs were incubated with different quantities of EELPH and incubated for 2 h at 37°C. The samples were then centrifuged at 4000 rpm for 5 min at 4 °C. The absorbance of each supernatant was determined at 545 nm. The RBCs were incubated with 0.5 w/v% Triton X-100 and PBS (pH 7.4) as positive and negative controls, respectively [51, 52]. Percentage (%) hemolysis was calculated using the following equation:

$$\%\;Hemolysis=\frac{absorbance\;sample-absorbance\;negative\;control}{absorbance\;positive\;control-absorbance\;negative\;control}*100$$ (3)

#### 2.2.10. Shelf life stability study

The optimized EELPH were kept at 4°C for 6 months. PS, ξ, as well as EE% were evaluated after 1, 3 and 6 months as previously described.

#### 2.2.11. *In vitro* cellular uptake study of DiI-labelled ELPH

*2*.*2*.*11*.*1*. *In vitro MTT cytotoxicity assay*. J774 macrophage cells (catalogue number ATCC^®^ TIB-67^™^) were purchased from ATCC, UK. Cells were deprived from reticulum cell sarcoma of BALB/cN mice. J774 macrophage cells were seeded in 96-well plate at a density of 10 K/ well in RPMI media and incubated overnight before being treated with the optimized ELPH or EELPH at serial drug concentrations ranging from 0.01–100 μM. Untreated cells or cells treated with the same concentration range of sodium lauryl sulfate were used as negative and positive control respectively. The *in vitro* cytotoxicity was assessed by MTT assay after 48 h of incubation, media was aspirated and cells were incubated with 120 μL of MTT solution at 37°C and 5% CO_2_. After 4 h, the formed formazan crystals were dissolved in 200 μL of DMSO and the plate read at 570 nm using FLUO star OPTIMA plate reader (BMG Labtech) [53]. The results were expressed as the percentage cell survival and calculated using the following equation:

$$\text{\%}\;\text{Cell}\;\text{survival}=\frac{\text{A}570\;\text{nm}\;\text{of}\;\text{treated}\;\text{cells}}{\text{A}570\;\text{nm}\;\text{of}\;\text{untreated}\;\text{control}\;\text{cells}}*100$$ (4)

*2*.*2*.*11*.*2 Determination of cellular uptake by confocal laser scanning microscope*. J774 macrophage cells were seeded onto sterile glass coverslips in 24-well plate at density 50 K cells/ well in RPMI media overnight. After incubation with 50 nM of the optimized DiI-labelled EELPH for 4 and 24 h, the cells were washed by PBS (pH 7.4) and fixed with 200 μL of 4% PFA for 15 min at room temperature. Subsequently, nucleus was counterstained with DAPI and the coverslips were mounted on glass slides by VectaShield^™^ mounting media. Cells were visualized by confocal laser scanning microscope (CLSM) (Carl Zeiss Microscopy GmbH, Germany) [54].

*2*.*2*.*11*.*3 Determination of cellular uptake by flow cytometry*. The cellular uptake of DiI-labelled ELPH and EELPH was quantified using flow cytometry (BD FACS Calibur^™^ flow cytometer, BD Biosciences). An overnight seeded J774 macrophage cells in 24-well plate at density 50 K cells /well were incubated with two different concentrations of the labelled optimized ELPH or EELPH (25 and 50 nM) for 4 and 24 h. Consequently, cells were washed twice with PBS, trypsinized and centrifuged at 1750 rom for 3 min at 4 °C. The collected cells were re-suspended into 200 μL of PBS. The uptake study was conducted at 10 K gated cells by quantifying the fluorescence using FL-2 detector and the obtained data was analysed using FlowJo software [55].

#### 2.2.12. Statistical analysis

Three replicates were done for each experiment and the recorded results were the mean ± SD. For comparing two variables student t-test was applied. For comparing different parameters between groups one-way analysis of variance (ANOVA) was applied followed by Tukey HSD test. All the analyses were performed using SPSS 18 (Chicago, USA) and differences were considered significant at probability (*p*) value *<0*.*05*.

## 3. Results and discussion

Designing nanocarriers capable of entrapping hydrophilic drugs in matrices with a delayed degradation, high encapsulating abilities and protected by a stabilizing coat to evade the immune system has attracted various researches in the last decade giving birth to the new progeny of LPH [56]. The molecular lipid barricade has to be optimized for adjusted size and noticeable prolonged release. In this context we aimed to prepare ELPH with high drug EE% and PS suitable for liver targeting of E, adopting a three steps study: 1) an optimization study to achieve the QTPP required for ELPH [57]; 2) a release experiment for the suggested ELPH by the model and finally 3) the effect of vitamin E coating on the optimized LPH physicochemical characteristics.

### 3.1. Optimization of ELPH using BBD

Generally, the physicochemical properties of nanocarriers affect their intracellular internalization and their subsequent therapeutic applications [58]. In this context, response surface methodology, using BBD, was constructed to ascertain the effect and interactions of different CPPs on PS (Y1) and EE% (Y2) (Table 2). According to the highest R^2^ and the lowest PRESS values, quadratic model was selected as the best fit statistical model for both PS and EE% responses (S1 and S2 Tables).

#### 3.1.1. Effect of different CPPS on Y1 and Y2

Table 2 shows that the fabricated ELPH had PS ranging between 125–310 nm, while drug EE % varied between 34.09 to 78.59%. All formulae showed a PDI less than 0.25 with a unimodal distribution. The effects of various significant CPPs on PS and EE% were described according to the following equations after omitting the non-significant terms:

Y1=+162+18A+13.17B+19.33C-64.67D+13.75AC-15.00BC+56.46D² (5)

Y2=+69.22+2.52A+2.00B+16.63C+5.16D-11.27AB-5.07A²-8.94C² (6)

ANOVA (S3 and S4 Tables) of the data reveals the regression coefficients of all assessed CPPs have p-values <0.05 indicating their significant effect.

**Fig 1:**
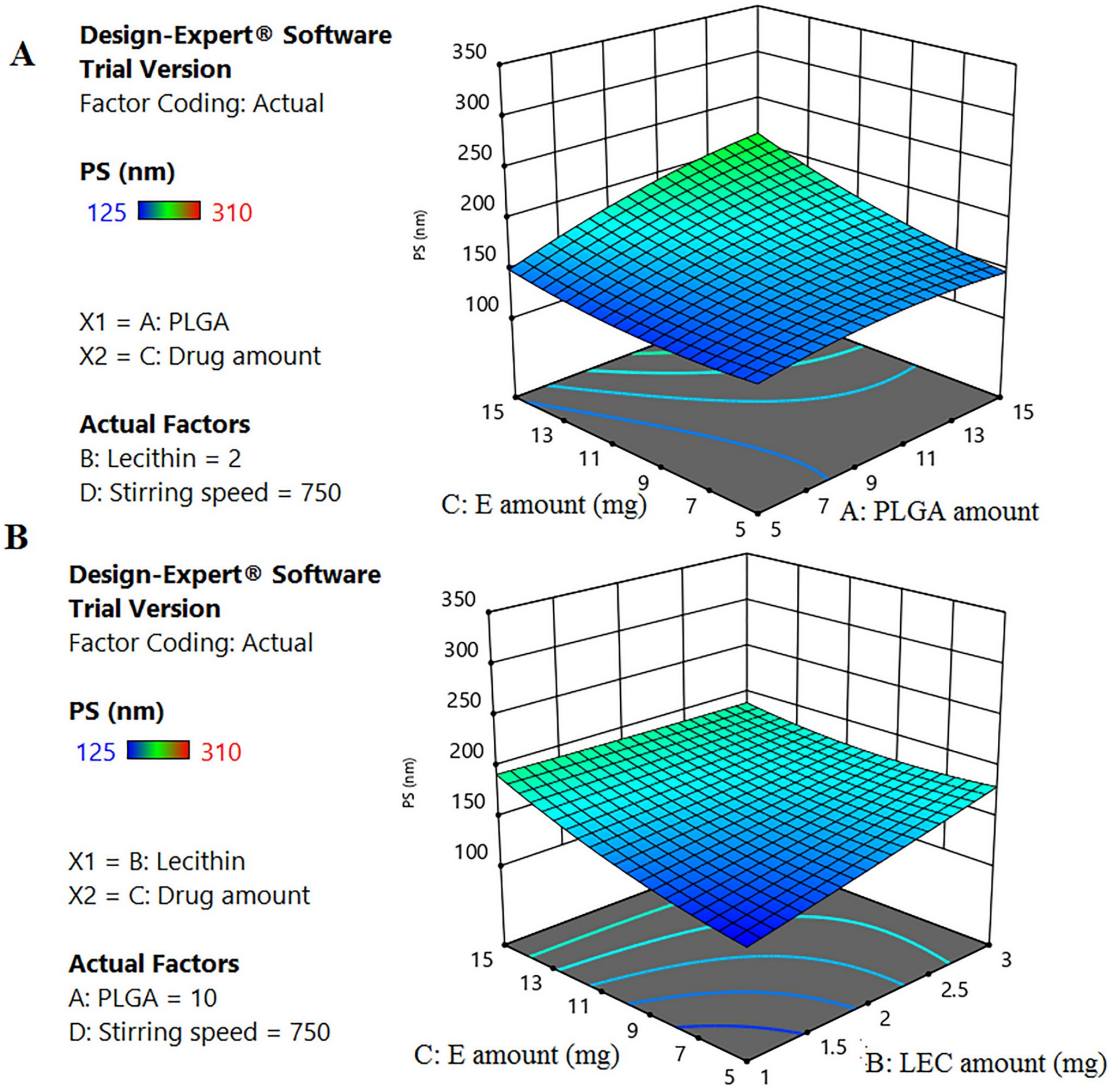
Response 3D plot for the significant parameters interaction on ELPH PS (Y1). Interaction of (AB) between PLGA and E (A). Interaction of (BC) between LEC and E (B). The positive interaction between PLGA and E indicates an enhanced effect of both variables on LPH PS. On the contrary, LEC-E interaction shows an antagonistic effect on PS.

According to Eq (5) and Fig 1, it is clear that ELPH PS was positively correlated with ***PLGA (A)*, *lipid (B)*, *drug amounts (C) and AC interaction*** with higher coefficients of ***A and C***. The higher organic phase component amounts, the polymer and the drug, would increase the viscosity of this phase thus decreasing its evaporation rate producing larger PS [59]. Moreover, this thick solution would hinder the breakdown of the droplets into smaller particles, opposing the shear force impact of stirring [60]. Similar findings were previously reported [14, 61, 62]. The positive interaction between ***A and C*** indicates an enhanced effect of both variables on LPH PS producing larger nanoparticulates (Fig 1).

Likewise, the polymer and drug effects on PS, increasing LEC content, significantly, enlarges PS. However, **BC** interaction shows an antagonistic effect on PS (Fig 1). This might be due to the possible attraction between the cationic E and the anionic LEC producing more compact smaller particles [12]. In contrast, the ***stirring speed (D)*** shows a negative effect on PS. The mechanical and hydraulic shear generated by increasing the stirring speed produced ELPH with lower PS [63].

**Fig 2:**
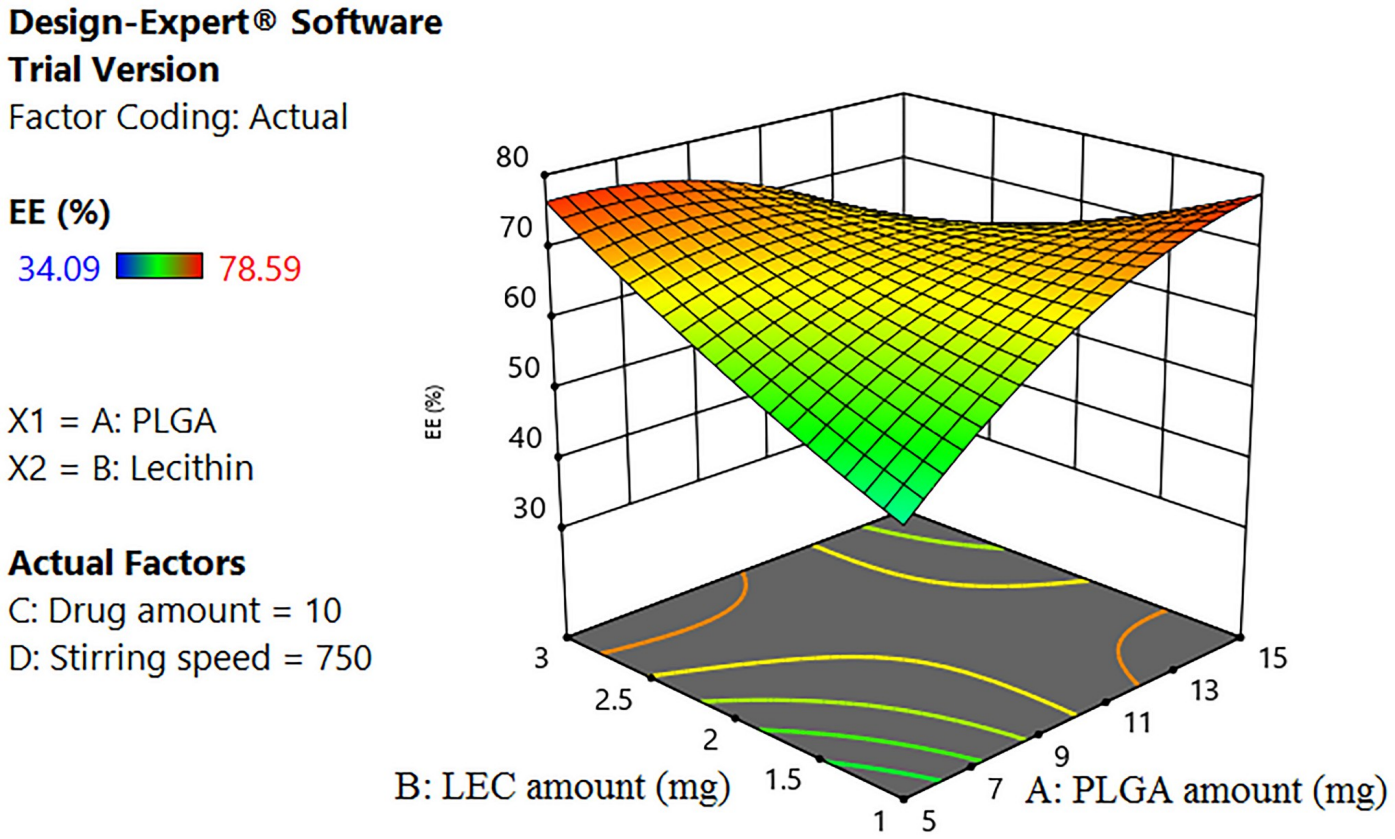
Response 3D plot for the interaction of (AB) between PLGA and LEC on ELPH EE% (Y2). The interaction between the polymer and lipid amounts has a negative influence on drug EE%.

Eq (6) shows a positive correlation between all factors effects and EE % with the highest one being that of the drug amount ***(C)***. Being a hydrophilic drug, increasing E amount enhanced its encapsulation. These results are in agreement with others who reported that EE% enhancement of glibenclamide was directly correlated to the increased drug content[64]. A direct positive effect was also found for ***LEC amount (B)*** on EE %, where the more the lipid content of the bilayer, the more its barrier effect is pronounced, hindering E diffusion from the polymer core [65]. An antagonistic effect of **AB** interaction between the polymer and lipid amounts on drug EE% was seen in Fig 2. Generally, the solubility of drug- polymer mixture into the melted lipid phase is a crucial parameter that determines the drug loading [66]. The miscibility of drug with the formulation components mainly depends on their mutual solubilities and polarities. By virtue of E hydrophilicity (log p -1.11) [67], the milieu is thought to be too hydrophobic to encapsulate it.

The EE% is significantly increased by rising the ***stirring speed (D)***. This might be attributed to the critical turbulent flow time when accompanied with high stirring speeds. In nanoprecipitation, nanoparticles are formed by interfacial phenomena due to the convection effects caused by interfacial turbulence. A physicochemical instability is produced by solvent transport and local regions of supersaturation are formed [68]. It might be assumed that turbulence, applied for a specified time, would enhance the evaporation of the organic solvent preventing drug leakage. Optimum flow rate was also reported during flash precipitation [69].

#### 3.1.2. Design space and optimization

The design space was plotted by overlapping different CPPs influence on CQAs contour plots to obtain QTPP. The yellow area represents the values of CPPs when optimized to fulfill QTPP criteria; minimum PS and maximum EE% (S1 Fig). Based on the high desirability and for model validation, three ELPH (F1-LEC, F2-LEC and F3-LEC) were selected and prepared as checkpoint. Table 2 depicts their compositions, sizes and EE %. It is worth mentioning that the linear correlation plots between experimental and predicted responses showed R^2^ value of 0.9932 and 0.9943 for Y1 and Y2 respectively indicating model suitability.

### 3.2. Evaluation of optimized ELPH and effect of lipid modification and vitamin E coating

Three optimized ELPH were characterized, displaying PS <200 nm, EE% values ˃75% and **PDI** values below 0.2. The **ξ** values were found to be from -14.51±1.85 to -25.51±2.62 depending on LEC and drug content (Table 3).

**Table 3:** Composition and characteristics of the optimized, lipid modified ELPH and vitamin E coated ELPH.

Code		Composition						Characteristics				
	Formula	PLGA (mg)	Lipid (mg)	E (mg)	Stirring Speed (rpm)	Lipid composition	LEC / CH or GMS ratio	PS (nm)^c^^,^^f^^,^ ^g^	PDI^c^^,^ ^f^	EE%^d^^,^ ^f^	ξ(mV)^c^^,^ ^f^^,^ ^g^	Drug loading (%)^e^^,^ ^f^^,^ ^g^
ELPH^[^^a^^,^ ^f^^]^	F1-LEC	5	2.85	13.33	900	LEC		122±5	0.176 ±0.01	81.25±2	-18.26±1	8.45±1
	F2-LEC	15	1	9.45	900			128.6±4	0.184±0.02	79.54±3	-14.51±2	6.13±0.3
	F3-LEC	5	2.93	9.45	1000			128±5	0.151±0.02	77.12± 2	-25.51±3	6.21±0.2
	F1-CH	5	2.85	13.33	900	LEC /CH	1:1.5	166.33±10*	0.154±0.01	79.89±2	-32.51±2*	8.36±0.11
	F2-CH	15	1	9.45	900			168.66±6*	0.139±0.02	78.73±3	-25.32±2*	6.02±0.12
	F3-CH	5	2.93	9.45	1000			160.33±9*	0.182±0.02	74.26±2	-42.88±1*	5.87± 0.14
	F1-GMS	5	2.85	13.33	900	LEC /GMS	1:1	176±8*	0.185±0.02	78.56±2	-38.17±1*	8.48±0.13
	F2-GMS	15	1	9.45	900			172.33±3*	0.173±0.02	79.70±2	-29.19±1*	6.02±0.2
	F3-GMS	5	2.93	9.45	1000			170±6*	0.167±0.02	79.23±1	-50.19±2*	6.05±0.2
EELPH^[^^b^^,^ ^f^^]^	F1-GMS/Vit E	5	2.85	13.33	900	LEC /GMS/vit E	1:1	190.66±4*	0.117±0.01	79.73±1	-30.68±2*	7.41±0.2*
	F2-GMS/Vit E	15	1	9.45	900			188.66±4*	0.143±0.02	80.47±2	-21.55±1*	5.49±0.2*
	F3-GMS/Vit E	5	2.93	9.45	1000			190 ±7*	0.187±0.02	76.11±2	-42.53±3*	5.30±0.4*

^a^ ELPH contains LEC, LEC-CH or LEC-GMS as a lipid shell

^b^ EELPH is vitamin E coated E loaded GMS LPH

^c^ Measured by DLS

^d^ Calculated as percentage of initial E added, determined directly by HPLC.

^e^ Calculated as percentage of entrapped E weight to the total ELPH or EELPH weight.

^f^ Results are expressed as mean ± SD (n = 3).

^g^ Statistical analysis was carried out using student’s T-test

*p<0.05.

Comparison between lipids modified LPH with LEC-based LPH.

Moreover, the **drug LC %** of the optimized formulae ranged from 6.13 to 8.45% (Table 3). This was consistent with previous literature that showed similar LC% for different drugs, when formulated as LPH, such as: ≈9% for amphotericin B [51], 1.8–9% with erlotinib [70] and 8–10% in case of etoposide [71]. It is to be pointed out that though the differences between LC % of the three ELPH (F1-LEC, F2-LEC and F3-LEC) were significantly different (*p> 0*.*05*), yet the values are not critical from a practical point of view.

#### 3.2.1. Effect of lipid modification on ELPH characteristics

Previous studies had outlined the effect of lipid composition on the physicochemical properties of LPH including drug release [72]. Others have reported that the drug content affected the drug release from LPH [73] and a third group proved a 7 day release for docetaxel sodium from LPH with LEC and GMS as lipid components [74].

Accordingly, various lipid mixtures were tested and tailored to control the drug release from the selected ELPH. Following a preliminary screening, optimized ELPH were prepared using LEC to CH in 1:1.5 ratio and LEC to GMS 1:1, while keeping the total lipid amount constant. These formulae were labeled F1-CH, F2-CH and F3-CH for CH modified systems or F1-GMS, F2-GMS and F3-GMS for GMS based ones (Table 3).

Increasing CH or GMS amounts significantly increased the PS of all the fabricated lipid modified ELPH ***(p<0*.*05)***. LPH prepared using lipid combinations were reported to be larger than those prepared using single ones [56]. By comparing the impact of CH and GMS modification on the PS, it could be concluded that GMS based LPH had significantly higher PS than their counterparts, CH based ones (p<0.05). This could be due to the larger molecular area of GMS (40 A°^2^) in comparison to CH (32 A°^2^) [75]. Similarly, the addition of either CH or GMS increased significantly the electronegativity of LPH (p<0.05). The higher absolute ξ values obtained with GMS, might be due to the possible free fatty acids of GMS [76]. In contrast, the incorporation of CH or GMS (Table 3) exhibited non-significant effect on both drug EE% and LC% whatever the composition of the LPH lipid shell (p>0.05).

### 3.3. Drug release study

The controlled release pattern in nanoparticulate systems is an essential prerequisite for efficient therapeutic outcomes [77], possibly by avoiding the undesirable circulation drug leakage [50]. A feature generally enhanced by LPH due to the barrier effect of the lipid shell [26].

**Fig 3:**
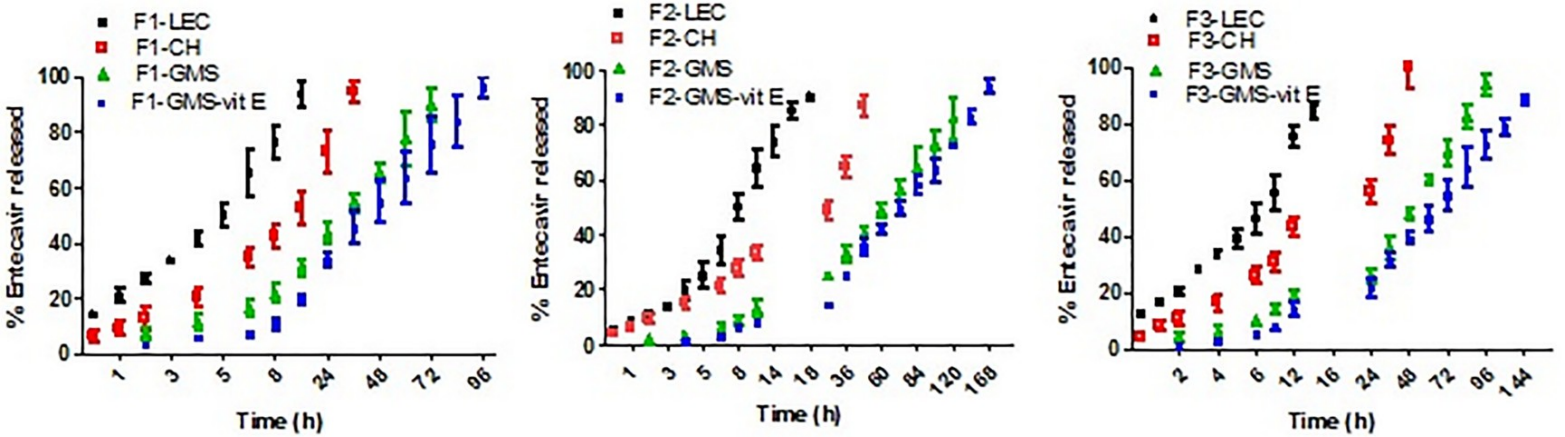
*In vitro* release profile of drug from different lipid polymer hybrid nanoparticles. *In vitro* E release from F1 prepared using LEC, LEC-CH, LEC-GMS and vitamin E coated LEC-GMS as lipid shell (A), F2 prepared using LEC, LEC-CH, LEC-GMS and vitamin E coated LEC-GMS as lipid shell (B), F3 prepared using LEC, LEC-CH, LEC-GMS and vitamin E coated LEC-GMS as lipid shell (C) after exposure to 50% final concentration rat serum. E release was measured by dialyzing each formula against PBS (pH 7.4). Drug concentration in the dialysate was quantified by HPLC. Data point represents mean and SD (n = 3).

All LPH formulations, prepared using LEC as the sole component of the lipid shell exhibited the same release pattern for 12–18 h (<24 h). (Fig 3A–3C) A release controlled by PLGA and the phospholipid. By applying the similarity factor calculations, F1-LEC and F3-LEC and F2-LEC vs. F3-LEC with respective (*f*2 = 44.55% and 46.84%) exhibited dissimilar release profiles. Accordingly, increasing the drug amount enhances the drug release. On the other hand, increasing polymer content prolonged E release. Additionally, the concomitant increase in PS observed with increasing PLGA content diminish the particles surface area available for drug release [78].

Modified lipid shell of ELPH by lipid variations may reduce the water penetration to the polymeric core, decreasing the polymer hydrolysis rate [19]. Generally, blending either CH or GMS with the LEC in the lipid shell during the fabrication of ELPH presented a more prolonged drug release than that of unmodified LEC based formulae. CH addition caused ~ 100% E release after 36, 48 and 42 h for F1-CH, F2-CH and F3-CH respectively (Fig 3A–3C) in comparison to less than 24 h with the phospholipid alone. Admixing GMS with LEC resulted in more drug release sustainment over 72, 120 and 96 h for the LPHs, F1-GMS, F2-GMS and F3-GMS respectively (Fig 3A–3C).

CH, an essential membrane-stabilizing lipid, has been widely explored in liposome formulations [79]. In addition to its stabilizing effect, CH was found to reduce water permeability through the lipid bilayer [72]. Similarly, the incorporation of GMS increased the LPH hydrophobicity, decreasing the interfacial area between the system and the dissolution medium [80]. Consequently, GMS significantly decreased the E release (p<0.05).

The more prolonged E release from GMS based formulae over CH based ones, could be correlated with the inverse relation between the drug-lipid solubility. Solubility studies show higher drug solubility in GMS, either alone or combined with LEC than in individual CH or CH- LEC mixture (S1 File, S5 Table). The high affinity of the drug to the lipid could hamper the partitioning of E to the dissolution medium as stated earlier [81]. Secondly, GMS was reported to be able of packing with LEC due to hydrophobic interaction between their respective hydrophobic parts and hydrogen bonds between their hydrophilic segments resulting in higher viscosity upon contact with water [82]. Thirdly, the higher PS, attained with GMS modified ELPH, could increase the diffusion path length [83]. in addition, the bigger PS with its smaller the surface area per unit volume available for release [84].

As hypothesized, combined coating of lipid shell (i.e LEC-GMS mixture) and the lipophilic vitamin E could offer more prolonged release prospects. Therefore, ELPH enriched with GMS were surface coated with vitamin E. Vitamin E, is a lipid soluble antioxidant with a partition coefficient 12.18. It exhibits a potent protective and repairing activity against peroxy radical induced biological membrane damage [85]. Moreover, vitamin E could be transferred physiologically from serum to the liver [86]. Hence, E GMS modified LPH were coated with vitamin E by physical adsorption producing EELPH. This could result in drug sustainment with possible stability and bioavailability improvement as described by [87]. Binding of vitamin E to the LEC in the lipid shell could occur via the interaction between the vitamin E phenoxyl hydroxyl group and the LEC phosphate and/or carbonyl group as well-described previously [88].

Successful coating due to the layer deposition on the fabricated systems was proved by a significant increase in PS (p<0.05). These results are in agreement with previous reports which demonstrated PS increase after coating of various NPs e.g.: TPGS coated polystyrene NPs, PEG and chitosan coated PLGA- Vitamin-E-TPGS NPs [87, 89]. The obtained PS and PDI values of the EELPH remained < 200 nm and <0.2 respectively. Moreover, the ξ. of EELPH formulae was significantly reduced if compared to naked uncoated formulae (p<0.05). This may be attributed to the ability of the coat to shift the shear plane of the diffusion layer to longer distance thus decrease the absolute value of ξ. relative to stern potential [87].

EE% in EELPH did not significantly changed (p> 0.05), while LC% exhibited a significant reduction due to mass increase (p<0.05) [70]. Similar results were obtained by Meng and coworkers [90] who explored the inverse relation between the number of coat layers on the PLGA and the loading efficiency of the encapsulated drug. However, the calculated loading efficiency values were still in acceptable range [70].

A more drug sustainment was noticed from all the vitamin E ELPH where about 100% of the drug was liberated after 96, 168 and 144 h with minimal burst effect from F1-GMS-vit E, F2-GMS-vit E and F3-GMS-vit E respectively (Fig 3A–3C). Vitamin E coat provided an additional partial lipophilic barricade on the LPH surface, offering steric force to E diffusion to the release medium [91]. It could be concluded that the approach of lipid coat tuning was efficient to control drug release for one week.

Therefore, formula vitamin E coated F2-GMS (will be referred as EELPH), showing the highest E retardation over one week, was selected for further characterization studies.

### 3.4. Morphological studies

**Fig 4:**
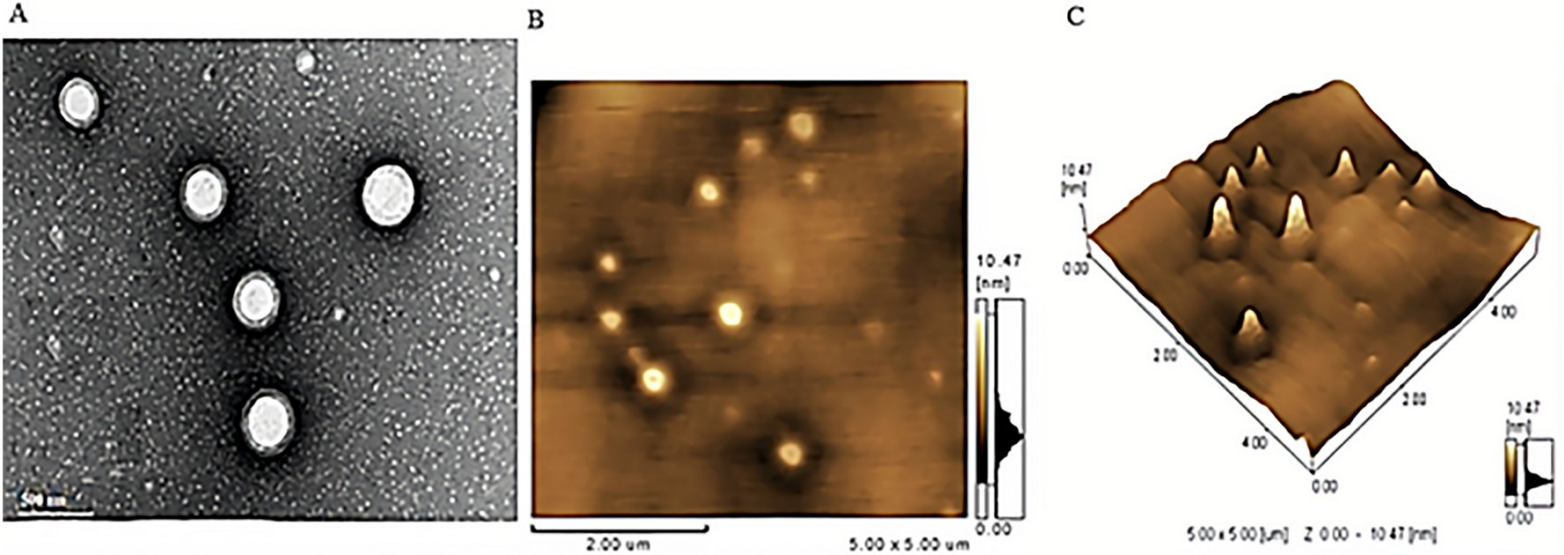
Morphological characterization of the optimized EELPH. Transmission electron micrograph (A), Atomic force micrograph planner view (B) and 3-D view (C) of EELPH. EELPH appeared as core-shell nanostructure with PS in consistency with DLS technique.

A representative TEM of the selected EELPH is illustrated in Fig 4A. Spherical non-aggregated particles with a PS in the range of 180–190 nm could be seen in the TEM which is consistent with the DLS measurements. The photomicrograph demonstrated the core-shell structure of the coated LPH. The white core in the center is the PLGA core while the gray ring around the polymer core is the coat “lipid shell” [70].

A core-shell structure of EELPH could be depicted from AFM Fig 4B. Moreover, the 3-D structure showed district particles with a PS in accordance with both TEM and DLS (Fig 4C).

### *3*.*5*. *In vitro* serum stability assay

**Fig 5:**
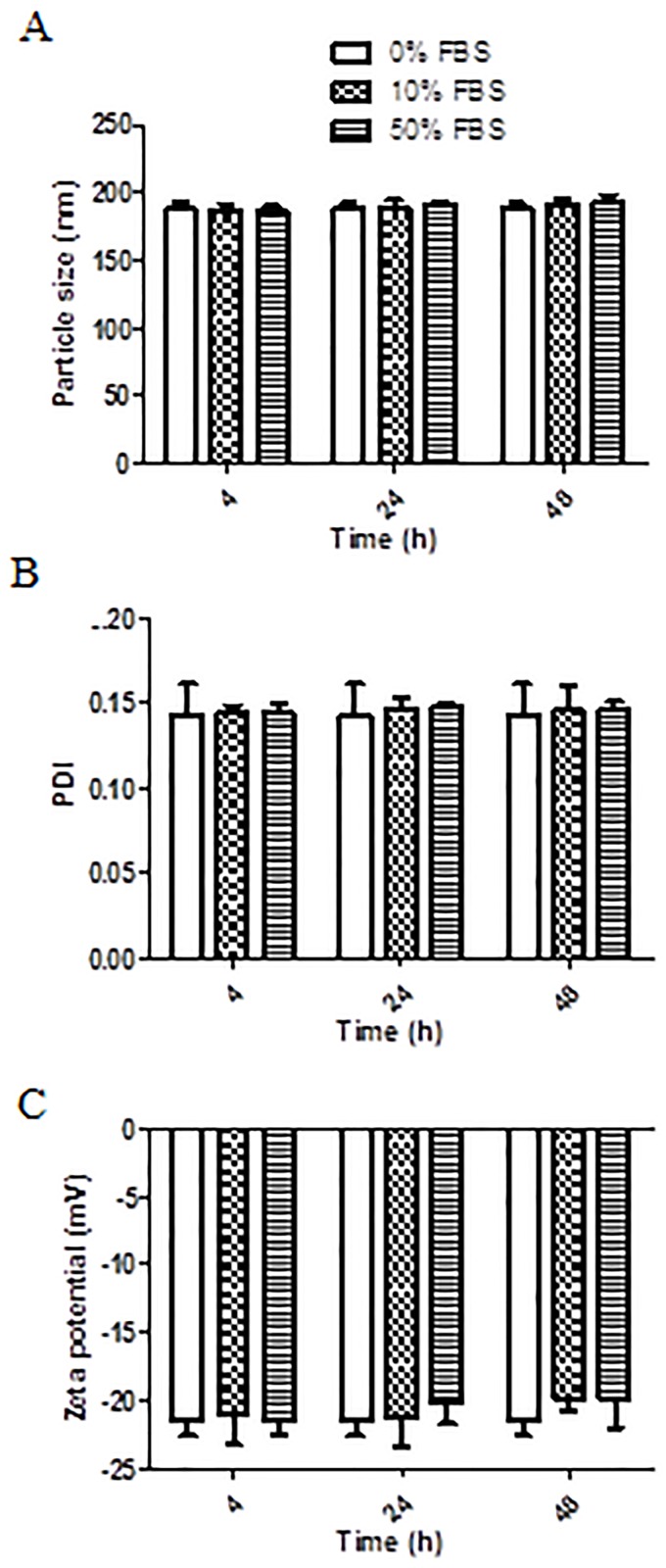
The effect of serum incubation on EELPH PS, PDI and ξ. The selected EELPH were incubated with 0%, 10% and 50% FBS for 4, 24 and 48h then PS (A), PDI (B) and ξ. (C) were measured using DLS as described. Data points represent mean and SD (n = 3). Statistical analysis was carried out using one-way ANOVA followed by Tukey HSD test and *P*<0.05 was considered significant. Serum protein had a non-significant effect on LPH PS, PDI or ξ.

The colloidal stability of the chosen EELPH was tested in 10% and 50% v/v FBS at 37 °C for 4 and 24 h. As shown in Fig 5A–5C, the formula was stable in 10% and 50% v/v FBS as proved by non-significant effect on PS, PDI and ξ at all tested time points (p> 0.05). The negligible effect of serum proteins could be ascribed by the electrostatic repulsion between the negatively charged LPH with the serum proteins [50, 92].

### 3.6. *In vitro* hemolytic assay

**Fig 6:**
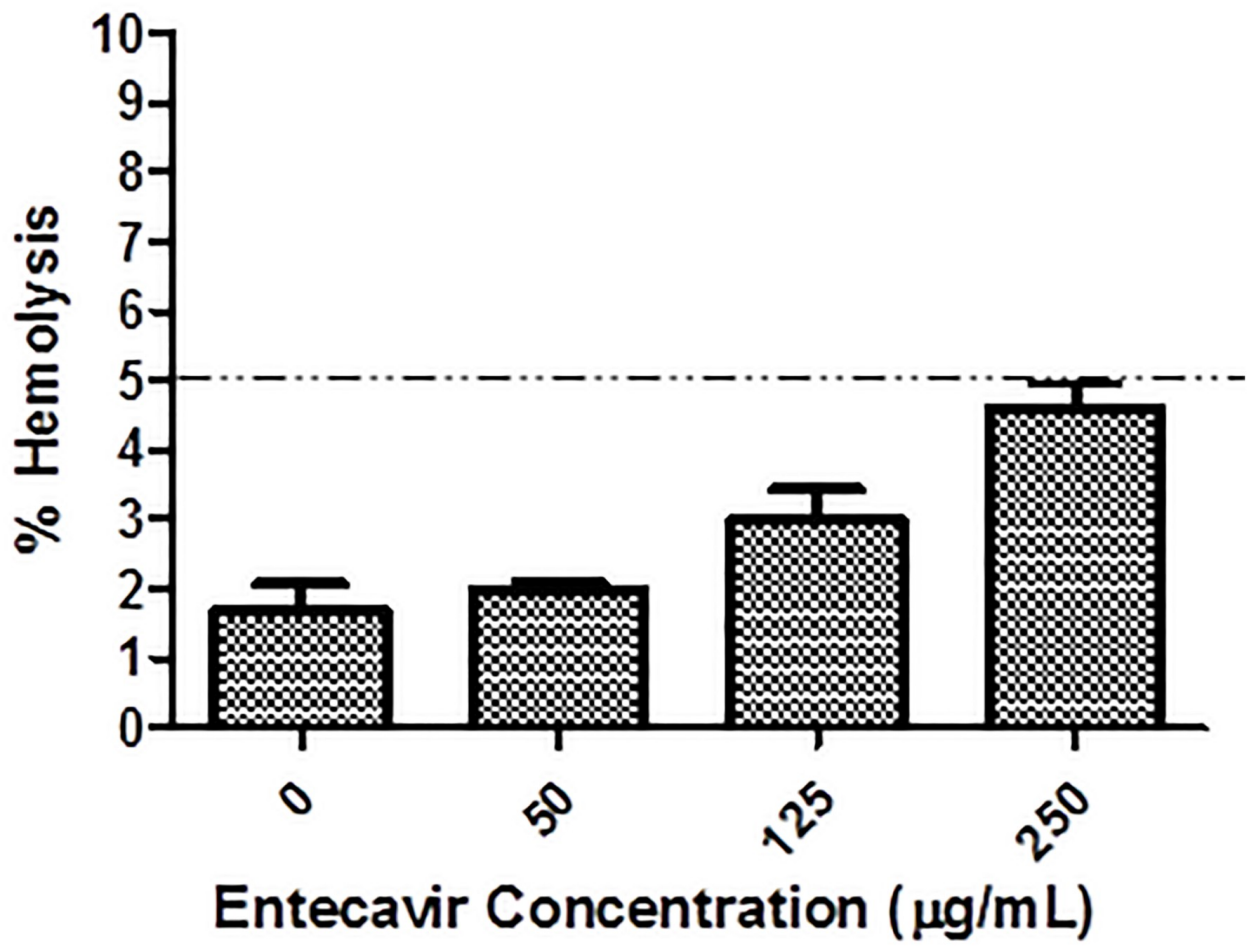
The *in vitro* hemolysis assay of EELPH. Rat RBCs were incubated with EELPH at different E concentrations (0–250 μg/mL) for 2 h at 37°C. Positive and negative controls were 0.5 w/v% Triton X-100 and PBS (pH 7.4), respectively. Samples were centrifuged at 4000 rpm for 5 min at 4 °C and the absorbance of the released haemoglobin was determined at 545 nm. Results are expressed as mean ± SD (n = 3). The dotted line represents the acceptable hemolysis range.

The biocompatibility of the fabricated EELPH was assessed by the *in vitro* hemolytic assay. The hemolytic activity was found to be heightened by a concomitant increase in the EELPH concentration. However, the calculated % hemolysis was < 5% in all tested quantities (Fig 6). Previous reports indicated that the accepted limit of hemolysis is from 5% to 25% [93]. However, the new consensus ASTM E2524-08 -Standard test method for analysis of hemolytic properties of nanoparticles limited the threshold to 5% [94]. The safety of NPs coated with vitamin E was reported by many previous studies [94, 95].

### 3.7. Shelf life colloidal stability study

The selected formula kept its original color with no phase separation or turbidity. No significant change (*p>0*.*05*) in the PS, PDI, ξ .or EE% was seen after 1, 3 and 6 months storage at 5°C at different time intervals (Table 4). It is worth noting that as stated earlier, following storage of LPH nanoparticles at climatic conditions of room temperature (25°C) over a period of 6 months, dramatic changes in the physicochemical properties of the hybrid particles, aggregates formation, degradation and possible corruption were noticed, signifying the necessity of keeping the storage temperature of LPH at 5°C [96].

**Table 4:** Characteristics of the selected EELPH at various time intervals following storage at 5°C over a 6 months period.

Parameter	Freshly prepared	Storage at 5 °C		
		1 month	3 month	6 month
PS (nm) ^a^^,^ ^c^^,^ ^d^	188.66±4	185.26±5	183.58±1.5	185.57±1
PDI ^a^^,^ ^c^^,^ ^d^	0.143±0.02	0.143± 0.01	0.155±0.01	0.148±0.01
ξ. (mV) ^a^^,^ ^c^^,^ ^d^	-21.55±1	-20.69± 2	-20.96±1	-20.79±2
EE% ^b^^,^ ^c^^,^ ^d^	80.47±2	80.57±2	79.71±1	81.51±2

^a^ Measured by DLS.

^b^ Calculated as percentage of initial E added, determined directly by HPLC.

^c^ Expressed as mean ± SD (n = 3).

^d^ Statistical analysis was carried out using one-way ANOVA followed by Tukey HSD test and *P*<0.05 was considered significant.

### 3.8. The effect of vitamin E coating on the cellular uptake of ELPH

**Fig 7:**
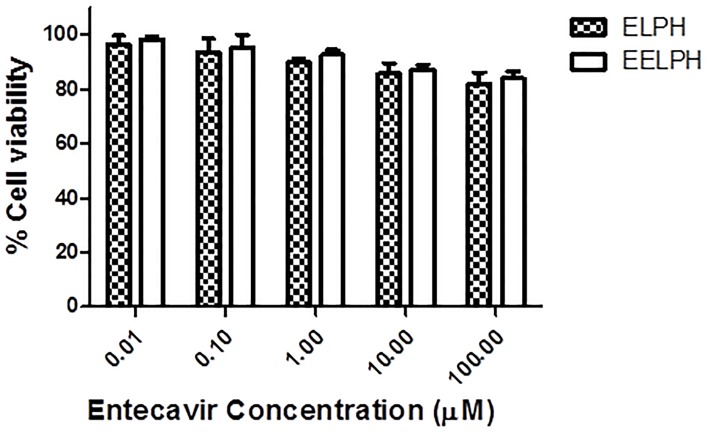
Cell viability assay of ELPH and EELPH after incubation for 48h. J774 macrophage cells were incubated with either ELPH or EELPH at increasing E concentrations (0.01–100 μM). Cells viability were assessed by MTT assay and results are presented as a percentage of the viable cells to the control untreated cells. Both tested formulae showed highe cell viability at all tested E concentrations. Data points are expressed as mean ± SD (n = 5).

High cell viability exceeding 80% was obtained up to a concentration equivalent to 100 μM E for the tested ELPH with or without vitamin E coating (Fig 7). The viability of cells treated with either ELPH or EELPH was significantly higher than that treated with sodium lauryl sulfate at the same concentration (P< 0.05).

**Fig 8:**
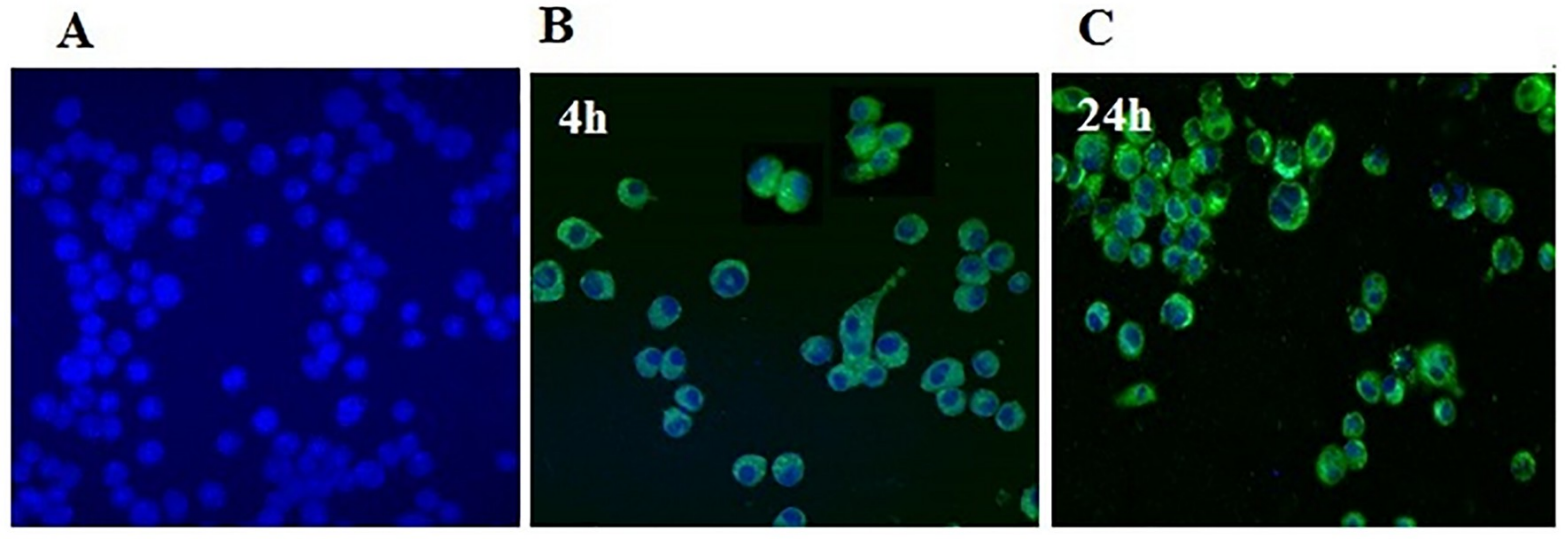
*In vitro* intracellular uptake of EELPH in J774 by confocal laser scanning microscopy. EELPH uptake was assessed by incubating J774 macrophage cells with 50 nM DiI-labelled EELPH for 4 h and 24. Untreated J774 cells appeared as blue due to the staining of the nucleus by DAPI (A), J774 cells treated with 50 nM DiI-labelled EELPH for 4 h (B) and 24 h (C). The uptake was confirmed by green fluorescence inside the cells while the cell nuclei appeared as blue due to the counterstaining with DAPI. The fluorescence intensity was increased in time-dependant manner.

Meaningful association between the *in vitro* phagocytosis of delivery nanoplatforms by macrophage and the *in vivo* liver retention is well-addressed [52]. In light of this, internalization of DiI-labelled EELPH (50 nM) in J774 cells was investigated qualitatively by CLSM. Due to the crucial potential of incubation time, boosting the cellular uptake efficiency [97], the cells were treated with coated LPH, considering two incubation times (4h and 24h). The DiI-labelled EELPH displayed green fluorescence, while J774 cells’ nuclei, stained with DAPI, showed blue fluorescence. Expectedly, enhanced time-dependent fluorescence intensity in the cytoplasm of J774 cells was exhibited (Fig 8), indicating meaningful retention efficiency in the cells and subsequent therapeutic efficacy of E [70].

**Fig 9:**
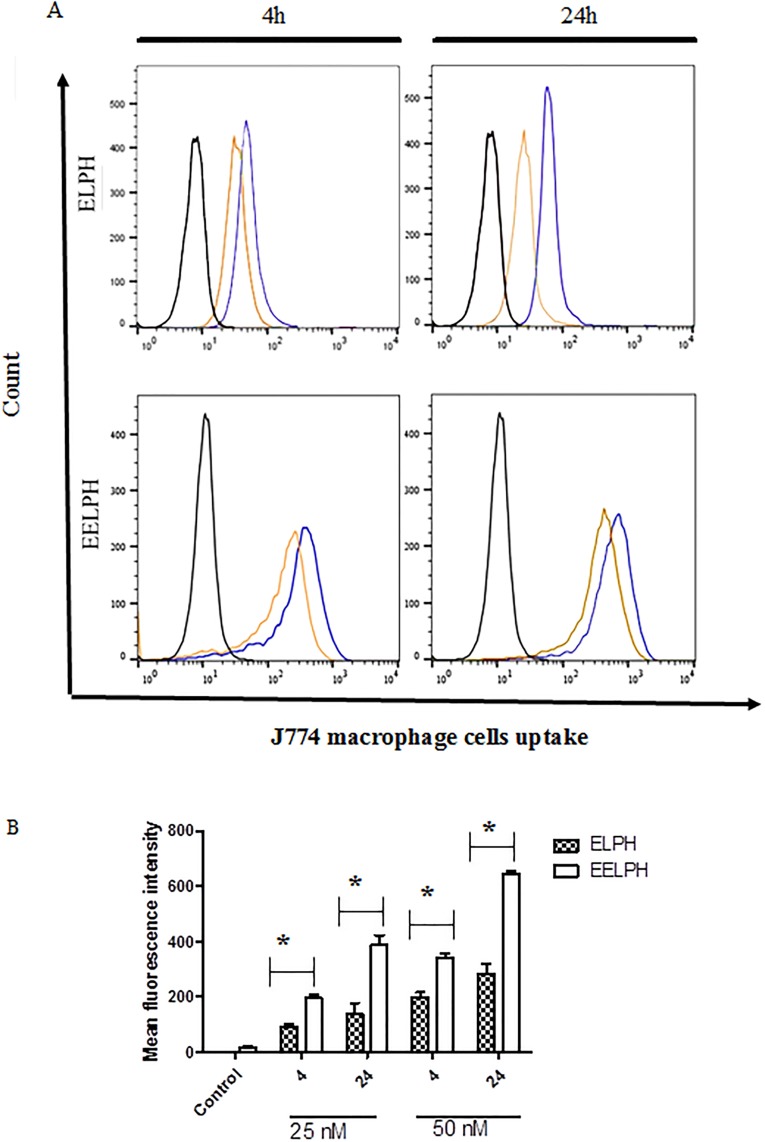
Intracellular uptake of ELPH and EELPH in J774 macrophage cells by flow cytometry. Cells were incubated with DiI-labelled ELPH or EELPH at 25 (brown) or 50 nM (blue) for 4 h or 24 h. Flow cytometry histogram for uptake of DiI-labelled ELPH and EELPH (A). Cellular uptake was quantified by mean florescence intensity (MFI) using flow cytometry and FL-2 detector (B). LPH uptake was higher for EELPH than ELPH and was shown to be dose- and time-dependent. Data points represent mean and SD (n = 3). Statistical analysis was carried out using one-way ANOVA followed by Tukey HSD test and *P*<0.05 was considered significant.

The intracellular internalization of DiI-labelled EELPH was further quantitatively assessed by flow cytometry. Moreover, in order to inspect the effect of vitamin E coating on the cellular uptake, cells were incubated with either EELPH or its uncoated counterpart ELPH (F2-GMS). Control J774 cells, without treatment, displayed auto fluorescence that was highly intensified following treatment of cells with DiI-labelled ELPH or EELPH in both time and concentration-dependent manners (Fig 9A).

As displayed in Fig 9B, increasing the concentration of either ELPH or EELPH from 25 to 50 nM yielded ≈1.5–2 fold increase in the mean fluorescence intensity (MFI) after 4 h. Meanwhile prolonging the incubation period to 24 h demonstrated ≈2 fold in the measured MFI. In addition, EELPH exhibited a significant 1.7–2 fold increase in the measured MFI values over the uncoated one. The presence of vitamin E significantly improved the cellular uptake efficiency by the macrophages (p< 0.05). This could be attributed to the potential ability of vitamin E to stimulate the phagocytic activity of macrophage as immunomodulatory to improve the phagocytic activity of macrophage as mentioned early [41].

## 4. Conclusion

HBV infection was considered as a major cause of death worldwide, due to the risk of cirrhosis, hepatocellular cancer, portal hypertension and liver failure. E monotherapy have been suggested for 48 weeks. Hence, the study offers a long-acting parenteral LPH of E. A platform, which combines the benefits of both polymeric and lipid carriers. The lipid shell was modulated with GMS to extend the drug release followed by Vitamin E coating to enhance liver targeting with more hydrophobic barriers for drug liberation. The optimized vitamin E coated LPH with LEC-GMS shell displayed favorable PS (188.66 nm) for passive targeting, an E entrapment of 80.47% and sustained release for one week. In addition, ELPH coated with vitamin E proved an increased in vitro macrophage retention, in comparison to the uncoated ones. In vivo antiviral activity should be considered in the future.

## Supporting information

S1 Fig Overlay plots depicting the design space region for the optimized ELPH. The design space was plotted by overlapping different CPPs influence on CQAs contour plots to obtain QTPP. The yellow area represents the values of CPPs when optimized to fulfill QTPP criteria; minimum PS (< 200 nm) and maximum EE%.
(TIF)

S1 File (DOCX)

S1 Table Model summary statistics for particle size (Y1). (DOCX)

S2 Table Model summary statistics for entrapment efficiency (Y2). (DOCX)

S3 Table ANOVA of the obtained data from BBD for the particle size of ELPH. (DOCX)

S4 Table ANOVA for the encapsulation efficiency for ELPH. (DOCX)

S5 Table Solubility of E in the utilized individual lipids and their combinations. (DOCX)
