# Supplementary figures and images for: An integrated vitamin E-coated polymer hybrid nanoplatform: A lucrative option for an enhanced in vitro macrophage retention for an anti-hepatitis B therapeutic prospect
Source: PLoS One. 2020 Jan 10;15(1):e0227231. doi: 10.1371/journal.pone.0227231 (PMC6953793; doi:10.1371/journal.pone.0227231)

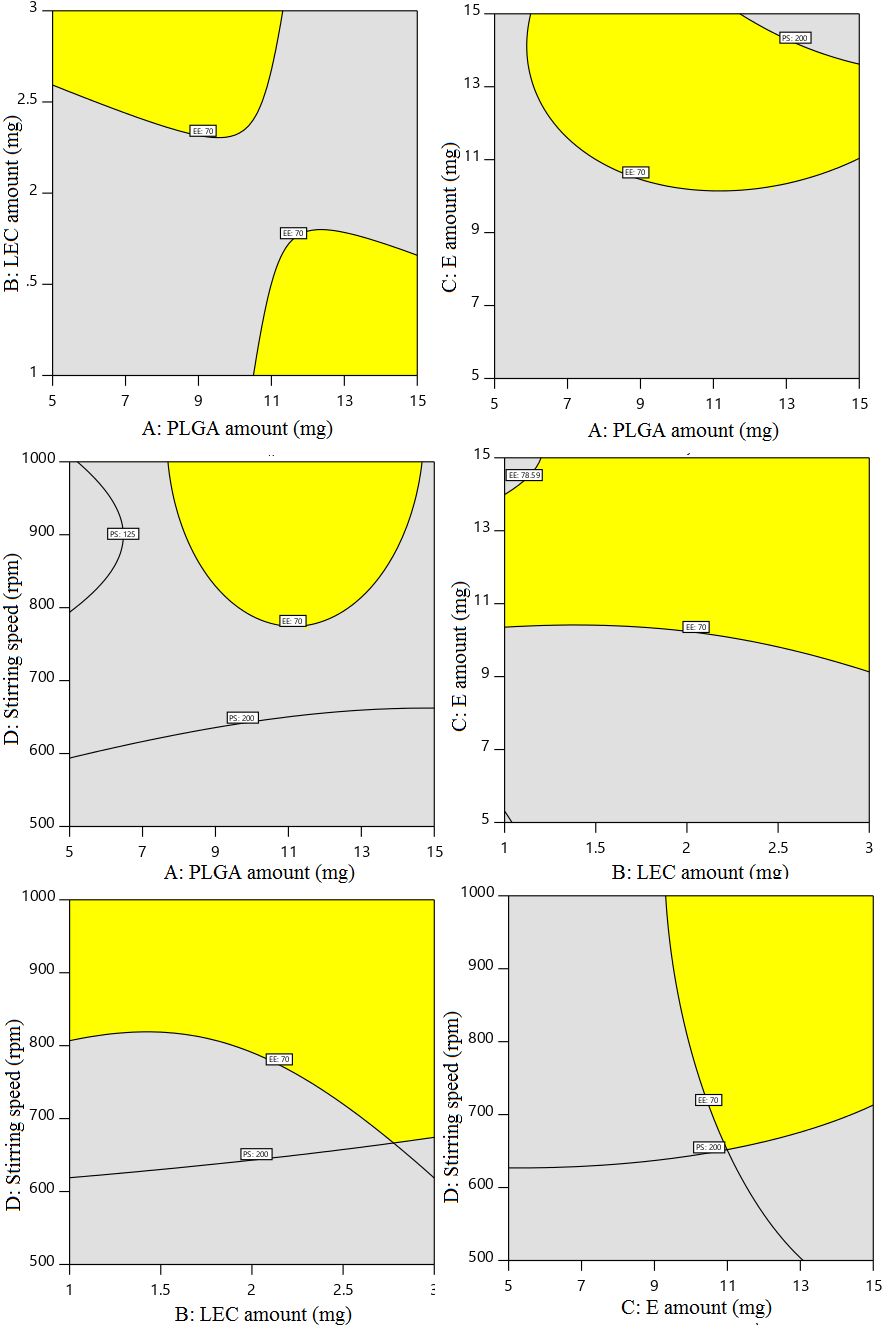

Supplement: S1 Fig — The design space was plotted by overlapping different CPPs influence on CQAs contour plots to obtain QTPP. The yellow area represents the values of CPPs when optimized to fulfill QTPP criteria; minimum PS (< 200 nm) and maximum EE%. (TIF) [file pone.0227231.s001.tif]
