# Supplementary material for: An integrated vitamin E-coated polymer hybrid nanoplatform: A lucrative option for an enhanced in vitro macrophage retention for an anti-hepatitis B therapeutic prospect
Source: PLoS One. 2020 Jan 10;15(1):e0227231. doi: 10.1371/journal.pone.0227231 (PMC6953793; doi:10.1371/journal.pone.0227231)
Supplement: S1 File — (DOCX) [file pone.0227231.s002.docx]

**Supplementary method**

1. **Determination of entecavir solubility in different lipids**

A known amount of entecavir (E) (10 mg) was placed in tightly closed bottles with 200 mg of different melted lipids namely; lecithin (LEC), cholesterol (CH) and glyceryl monostearate (GMS) either one lipid at a time or with different combinations of LEC with CH or GMS. Solubility study in LEC and GMS was conducted in a thermostatically controlled water bath maintained at 70°C ± 0.5 (above the melting point of both lipids). On the contrary, E-CH solubility study was performed into a thermostatically controlled silicone oil bath maintained at 150 °C ± 0.5 to ensure CH melting. The mixtures were kept at the required temperatures for one h. Subsequently, different aliquots of molten lipids were added at the specified temperature with constant stirring until the formation of a clear solution. Finally, the total volume of lipids producing a visually clear solution was recorded.
