# Supplementary material for: An integrated vitamin E-coated polymer hybrid nanoplatform: A lucrative option for an enhanced in vitro macrophage retention for an anti-hepatitis B therapeutic prospect
Source: PLoS One. 2020 Jan 10;15(1):e0227231. doi: 10.1371/journal.pone.0227231 (PMC6953793; doi:10.1371/journal.pone.0227231)
Supplement: S1 Table — (DOCX) [file pone.0227231.s003.docx]

**Supplementary tables**

**Table S1: Model summary statistics for particle size (Y1).**

| **Source** | **Standard Deviation (SD)** | **R²** | **Adjusted R²** | **Predicted R²** | **PRESS** |  |
| --- | --- | --- | --- | --- | --- | --- |
| Linear | 33.97 | 0.6864 | 0.6341 | 0.5269 | 41791.57 |  |
| 2FI | 37.52 | 0.7132 | 0.5539 | 0.1312 | 76748.88 |  |
| **Quadratic^[a]^** | **11.18** | **0.9802** | **0.9604** | **0.9087** | **8061.60** | **Suggested** |
| Cubic | 12.24 | 0.9898 | 0.9525 | 0.3128 | 60705.12 | Aliased |

**^[a]^**Adequate precision equals 22.71 and coefficient of variation (C.V.) % is 6.02
