# Supplementary material for: An integrated vitamin E-coated polymer hybrid nanoplatform: A lucrative option for an enhanced in vitro macrophage retention for an anti-hepatitis B therapeutic prospect
Source: PLoS One. 2020 Jan 10;15(1):e0227231. doi: 10.1371/journal.pone.0227231 (PMC6953793; doi:10.1371/journal.pone.0227231)
Supplement: S2 Table — (DOCX) [file pone.0227231.s004.docx]

**Table S2: Model summary statistics for entrapment efficiency (Y2).**

| **Source** | **Standard Deviation (SD)** | **R²** | **Adjusted R²** | **Predicted R²** | **PRESS** |  |
| --- | --- | --- | --- | --- | --- | --- |
| Linear | 7.68 | 0.7264 | 0.6808 | 0.5980 | 2081.48 |  |
| 2FI | 6.79 | 0.8398 | 0.7508 | 0.5897 | 2124.65 |  |
| **Quadratic^[a]^** | **3.16** | **0.9730** | **0.9459** | **0.9195** | **416.79** | **Suggested** |
| Cubic | 4.06 | 0.9809 | 0.9109 | 0.8028 | 1020.85 | Aliased |

**^[a]^** Adequate precision equals 19.83 and coefficient of variation (C.V.) % is 5.01
