# Supplementary material for: An integrated vitamin E-coated polymer hybrid nanoplatform: A lucrative option for an enhanced in vitro macrophage retention for an anti-hepatitis B therapeutic prospect
Source: PLoS One. 2020 Jan 10;15(1):e0227231. doi: 10.1371/journal.pone.0227231 (PMC6953793; doi:10.1371/journal.pone.0227231)
Supplement: S3 Table — (DOCX) [file pone.0227231.s005.docx]

**Table S3: ANOVA of the obtained data from BBD for the particle size of ELPH.**

| **Source** | **Sum of Squares** | **df^[a]^** | **Mean Square** | **F-value** | **p-value^[b]^** |
| --- | --- | --- | --- | --- | --- |
| Model | 86585.99 | 14 | 6184.71 | 49.45 | < 0.0001^*^ |
| A-PLGA | 3888.00 | 1 | 3888.00 | 31.09 | < 0.0001^*^ |
| B-LEC | 2080.33 | 1 | 2080.33 | 16.63 | 0.0011^*^ |
| C-E amount | 4485.33 | 1 | 4485.33 | 35.87 | < 0.0001^*^ |
| D-Stirring speed | 50181.33 | 1 | 50181.33 | 401.26 | < 0.0001^*^ |
| AB | 529.00 | 1 | 529.00 | 4.23 | 0.0589 |
| AC | 756.25 | 1 | 756.25 | 6.05 | 0.0276^*^ |
| AD | 56.25 | 1 | 56.25 | 0.4498 | 0.5133 |
| BC | 900.00 | 1 | 900.00 | 7.20 | 0.0179^*^ |
| BD | 16.00 | 1 | 16.00 | 0.1279 | 0.7259 |
| CD | 110.25 | 1 | 110.25 | 0.8816 | 0.3637 |
| A² | 501.36 | 1 | 501.36 | 4.01 | 0.0650 |
| B² | 3.25 | 1 | 3.25 | 0.0260 | 0.8741 |
| C² | 491.90 | 1 | 491.90 | 3.93 | 0.0673 |
| D² | 20675.96 | 1 | 20675.96 | 165.33 | < 0.0001^*^ |
| Residual | 1750.83 | 14 | 125.06 |  |  |
| Lack of Fit | 1268.83 | 10 | 126.88 | 1.05 | 0.5250 |
| Pure Error | 482.00 | 4 | 120.50 |  |  |
| Cor Total | 88336.83 | 28 |  |  |  |

**^[a]^**Degree of freedom

**^[b]^** *p< 0.05
