# Supplementary material for: An integrated vitamin E-coated polymer hybrid nanoplatform: A lucrative option for an enhanced in vitro macrophage retention for an anti-hepatitis B therapeutic prospect
Source: PLoS One. 2020 Jan 10;15(1):e0227231. doi: 10.1371/journal.pone.0227231 (PMC6953793; doi:10.1371/journal.pone.0227231)
Supplement: S4 Table — (DOCX) [file pone.0227231.s006.docx]

**Table S4: ANOVA for the encapsulation efficiency for ELPH.**

| **Source** | **Sum of Squares** | **df^[a]^** | **Mean Square** | **F-value** | **p-value^[b]^** |
| --- | --- | --- | --- | --- | --- |
| Model | 5038.08 | 14 | 359.86 | 36.00 | < 0.0001^*^ |
| A-PLGA | 76.41 | 1 | 76.41 | 7.64 | 0.0152^*^ |
| B-LEC | 47.96 | 1 | 47.96 | 4.80 | 0.0459^*^ |
| C-Drug amount | 3317.69 | 1 | 3317.69 | 331.89 | < 0.0001^*^ |
| D-Stirring speed | 319.30 | 1 | 319.30 | 31.94 | < 0.0001^*^ |
| AB | 508.28 | 1 | 508.28 | 50.85 | < 0.0001^*^ |
| AC | 19.71 | 1 | 19.71 | 1.97 | 0.1820 |
| AD | 0.0380 | 1 | 0.0380 | 0.0038 | 0.9517 |
| BC | 35.16 | 1 | 35.16 | 3.52 | 0.0817 |
| BD | 6.60 | 1 | 6.60 | 0.6607 | 0.4299 |
| CD | 17.26 | 1 | 17.26 | 1.73 | 0.2099 |
| A² | 166.59 | 1 | 166.59 | 16.66 | 0.0011^*^ |
| B² | 13.28 | 1 | 13.28 | 1.33 | 0.2683 |
| C² | 518.89 | 1 | 518.89 | 51.91 | < 0.0001^*^ |
| D² | 31.76 | 1 | 31.76 | 3.18 | 0.0964 |
| Residual | 139.95 | 14 | 10.00 |  |  |
| Lack of Fit | 47.20 | 10 | 4.72 | 0.2036 | 0.9812 |
| Pure Error | 92.75 | 4 | 23.19 |  |  |
| Cor Total | 5178.03 | 28 |  |  |  |

**^[a]^**Degree of freedom

**^[b]^** * p< 0.05
