# Supplementary material for: An integrated vitamin E-coated polymer hybrid nanoplatform: A lucrative option for an enhanced in vitro macrophage retention for an anti-hepatitis B therapeutic prospect
Source: PLoS One. 2020 Jan 10;15(1):e0227231. doi: 10.1371/journal.pone.0227231 (PMC6953793; doi:10.1371/journal.pone.0227231)
Supplement: S5 Table — (DOCX) [file pone.0227231.s007.docx]

**Table S5: Solubility of E in the utilized individual lipids and their combinations.**

| **Lipid** | **LEC to GMS or CH ratio (w:w)** | **Amount of lipids required to dissolve 10 mg of E (g) ^[a]^** |
| --- | --- | --- |
| **LEC** | 1:0 | 0.24±0.036 |
| **GMS** | 0:1 | 0.4±0.05 |
| **LEC: GMS** | 1:1 | 0.69±0.04 |
| **CH** | 0:1 | 1.51±0.1 |
| **LEC:CH** | 1:1.5 | 0.97±0.02 |
|  |  |  |

**^[a]^** Results are mean of three determinations ± standard deviation (SD).
